# Alveolar rhabdomyosarcoma with multiple bone marrow metastases: a case report

**DOI:** 10.1093/bjrcr/uaaf066

**Published:** 2025-12-17

**Authors:** Deqing Song, Yining Xiang, Lu Shen, Lingling Song

**Affiliations:** Department of Radiology, Affiliated Hospital of Guizhou Medical University, Guiyang 550000, China; Department of Pathology, Affiliated Hospital of Guizhou Medical University, Guiyang 550000, China; Department of Radiology, Affiliated Hospital of Guizhou Medical University, Guiyang 550000, China; Department of Radiology, Affiliated Hospital of Guizhou Medical University, Guiyang 550000, China

**Keywords:** alveolar rhabdomyosarcoma, neoplasm metastases, magnetic resonance imaging, computed tomography, adolescent oncology

## Abstract

Rhabdomyosarcoma (RMS) is rare but is the most common soft tissue sarcoma in children and teenagers. Alveolar rhabdomyosarcoma (ARMS), a subtype of RMS, primarily affects teenagers aged between 10 and 25 years old and is associated with early lymphatic and hematogenous metastases. This case report describes a 13-year-old girl with ARMS in the right thigh, accompanied by multiple bone and bone marrow metastases. Contrast-enhanced CT/MRI revealed a mass with marked heterogeneous enhancement and an enhancing pseudocapsule, while pathological examination confirmed the diagnosis through immunohistochemical markers (MyoD1 (+), Myogenin (+), Desmin (+)). This case highlights the aggressive nature of ARMS and the importance of characteristic imaging findings.

## Introduction

Rhabdomyosarcoma (RMS) is a highly malignant soft tissue tumour that derives from rhabdomyocytes or primitive mesenchymal cells with the ability to differentiate into rhabdomyocytes, and it is most commonly found in children and teenagers. According to the 2020 World Health Organization classification, RMS is categorised into four subtypes: embryonal, alveolar, pleomorphic, and spindle cell/sclerosing RMS.^1^ Alveolar rhabdomyosarcoma (ARMS), accounting for 15%-20% of cases, is characterized by aggressive behaviour and early distant metastases.^2^ Computed tomography (CT) and magnetic resonance imaging (MRI) play a critical role in determining tumour extent and metastatic spread. We hope to improve understanding of ARMS by describing our own case and highlighting its clinical and imaging aspects.

## Case presentation

A 13-year-old girl presented with a two-month history of backache and a palpable right thigh lump. The laboratory examination revealed no evident abnormalities. During physical examination, a hard mass measuring 10.0 × 8.0 cm was found on the anterior area of the right thigh with no skin abnormalties.

### Imaging findings

**Figure 1.**
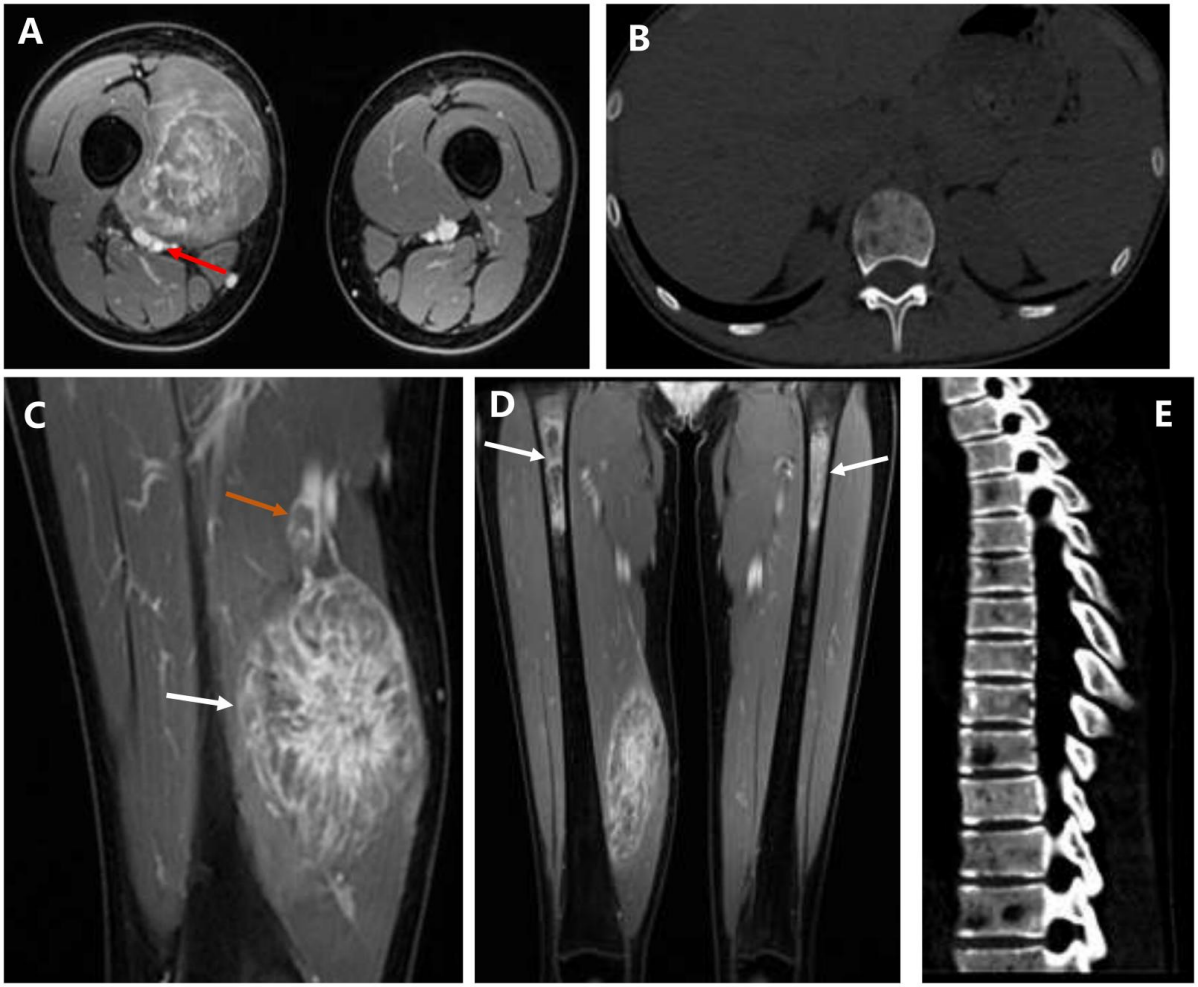
Contrast-enhanced MRI (A, C, D) and CT (B, E) of the ARMS. (A, C, D) Contrast-enhanced MR imaging showed marked heterogeneous enhancement of the mass in the right vastus medialis muscle, which manifested as a distinctive “chrysanthemum petal-like” morphology created by radiating enhancing septa. (A)The femoral artery posterior to the mass was displaced and closely abutted by the lesion (red arrow). (C) Sagittal MRI shows pseudocapsule enhancement at the edge of the mass (white arrow). An intraluminal filling defect demonstrating heterogeneous enhancement was identified within the femoral vein (orange arrow). (D) Coronal MRI showed multiple patchy abnormal enhancements in the bilateral proximal femoral diaphyses (white arrows). Plain CT scan of chest (B, bone window) and Sagittal reformatted CT scan images of the thoracolumbar spine (E, bone window) showed multiple lesions of low-density bone destruction in the thoracolumbar spine.

Contrast-enhanced MRI demonstrated a 6.1 × 4.6 × 10.0 cm heterogeneously enhancing mass in the right vastus medialis muscle, exhibiting marked heterogeneous enhancement (Figure 1A, C, and D). The pseudocapsule was noticeably enhanced. The femoral artery posterior to the mass was displaced and closely abutted by the lesion, but without definite radiologic evidence of invasion. In contrast, the femoral vein was infiltrated by the mass, resulting in an intraluminal filling defect that demonstrated heterogeneous enhancement. The mass displayed infiltrative growth along the muscle fibres towards the ends and was most visible in the muscle’s belly. The mass was either fusiform or elongated, with the longitudinal axis parallel to the muscle bundle.

Multiple patchy enhancing metastases were observed in the bilateral proximal femoral diaphyses (Figure 1D). The right-sided lesions showed predominant ring-like enhancement, whereas the left-sided lesions demonstrated heterogeneous enhancement. Plain CT scan of chest and Sagittal reformatted CT scan images of the thoracolumbar spine revealed multiple lytic lesions in the thoracolumbar spine (Figure 1B and E). Both the lymph nodes and the parenchymal organs were devoid of metastases.

### Pathological findings

**Figure 2.**
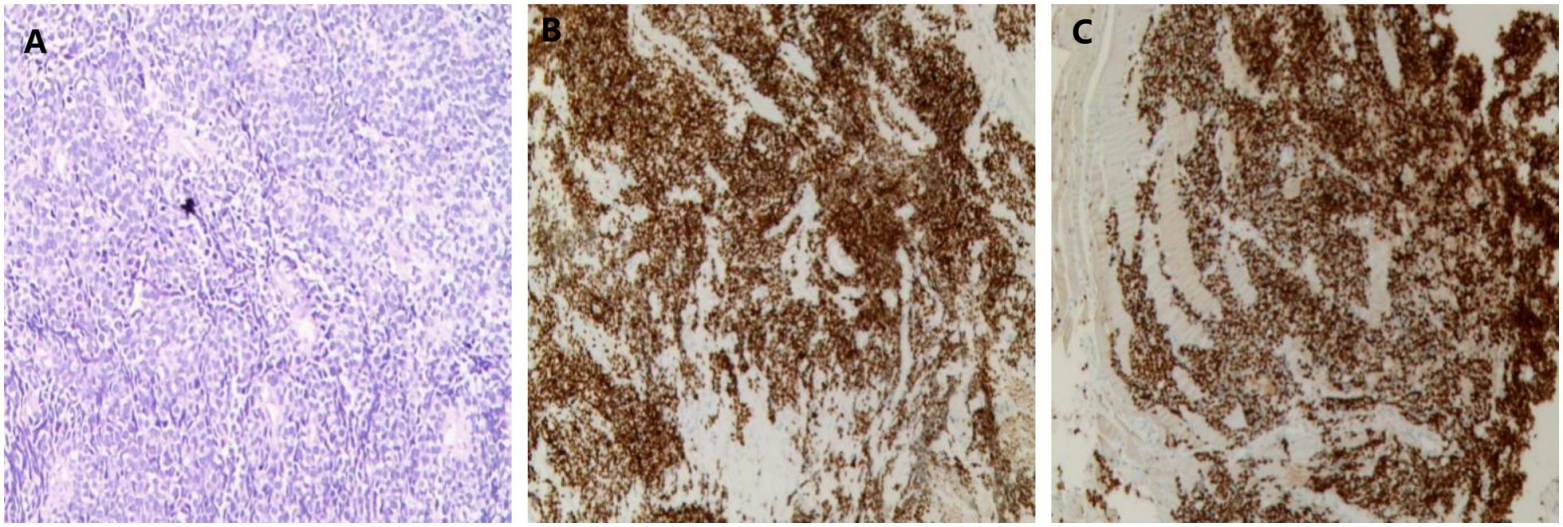
Histopathological results. (A) Hematoxylin-eosin (HE) staining (×200). The tumor cells were round or oval, with little cytoplasm and hyperchromatic nuclei. Immunohistochemistry positivity for MyoD1 (B) and Myogenin (C).

A puncture biopsy of the lumbar 3 vertebral lesion and right thigh mass was performed. Pathological examination revealed small round tumour cells with hyperchromatic nuclei and nuclear fission, arranged in alveolar patterns with fibrovascular septa (Figure 2A). Immunohistochemistry showed Vim (+), Desmin (+), MyoD1 (+), Myogenin (+), CD56 (+), S100 (partial+), CK (-), EMA (-), SMA (-), Caldesmon (-), CgA (-), Syn (-), CD99 (-), NKX2.2 (-), FLi1 (-), ERG (-), SOX10 (-), HMB45 (-), Melan-A (-), and Ki-67 (70-80%+) (Figure 2B and C). Fluorescence in situ hybridization (FISH) analysis excluded *EWSR1* rearrangement.

The patient was discharged against medical advice at the request of both the patient and the family, who declined any further treatment. The patient succumbed to the disease 6 months after discharge.

## Discussion

ARMS is a rare type of rhabdomyosarcoma. It is more frequent in individuals aged 10-25, with almost equal prevalence among males and females.^3^ The disease primarily affects the deep soft tissues of the extremities, followed by the head and neck, trunk, perineum, pelvis and retroperitoneum, with a few cases affecting subcutaneous tissues.^4^ The clinical manifestation of ARMS is a rapidly growing mass that may cause pain. The tumour is infiltrative with unclear boundaries. ARMS of the extremities has been reported to be more aggressive, with a higher incidence of atypical anatomical metastases. This malignant soft tissue tumour can spread through direct invasion, lymphatic or hematogenous metastasis.^5^ At the time of presentation, the patient had metastasised to many sites, supporting the literature’s claim of rapid ARMS progression.^2^ In terms of imaging characteristics, ARMS resembled general soft tissue malignant tumours, with an isodense or slightly low-density mass on CT scan. The MRI manifestations were generally equal or slightly low signal on T1WI, and moderate to obviously high signal on T2WI.^6^ The imaging hallmark of ARMS, marked heterogeneous enhancement, was observed in this case, which is consistent with prior reports.^5^^,^^7^ A distinctive “chrysanthemum petal-like” morphology, created by radiating enhancing septa, was observed in this specific case. While the descriptive term has been mentioned in the literature, it was not clearly demonstrated in the cases presented therein.^8^^,^^9^ Furthermore, it is worth noting that the lesion spread infiltratively along the muscle fibres to both ends. In this case, MRI demonstrated an enhancing pseudocapsule, a feature that may contribute to the radiographic assessment of soft tissue masses.

Pathologically, primitive small round tumour cells were observed to be arranged in nest-like and sheet-like patterns, forming characteristic alveoli-like structures, with fibrovascular septa separating the alveoli. In some areas, skeletal muscle differentiation was partially evident.^10^ Based on the amount of fibrovascular interstitium, three histological subtypes have been identified: classic, solid, and embryonic-alveolar mixed.^5^ ARMS predominantly expressed myogenic markers, including Desmin, Myogenin, and MyoD1. The tumour cells were strongly and diffusely positive for Myogenin, which aids in distinguishing ARMS from embryonal rhabdomyosarcoma. The case was positive for MyoD1, Myogenin, and Desmin, leading to the diagnosis of solid subtype ARMS.

## Conclusion

ARMS should be suspected when contrast-enhanced CT or MRI reveals a soft tissue mass with marked heterogeneous enhancement. Early pathological confirmation is critical for timely intervention.

## Learning points

- Marked heterogeneous enhancement on MRI is a key diagnostic clue for ARMS.
- Bone marrow metastases indicate advanced disease and poor prognosis.
